# The Treatment of Epilepsy

**Published:** 1895-12-14

**Authors:** 


### The Treatment of Epilepsy.

Few things have been more remarkable during the year which is passing away than the advance which has been made in the opinions alike of the medical profession and of the public with regard to the treatment of epilepsy, or more properly, of epileptics. In the early part of the century epilepsy was practically outside the domain of the physician, and a very large proportion of epileptics drifted into public or private lunatic asylums, where the only care taken of them was by the adoption of precautions intended to secure them against injury. They were liable to turn face downwards in bed during nocturnal fits, and so to suffocate themselves, and it was customary to chain them by one arm to the side of the bedstead in order to render such turning impossible. One result of this practice was that they were often chained for long periods and were rendered ferocious, like chained dogs. In those days, consequently, apart from the circumstance that some forms of epilepsy are apt to be followed by periods of subjugation to destructive impulses, the epileptic ward was the most dangerous place in a lunatic asylum, and the epileptics were the most dangerous class among the inmates. When released from chains, they were usually compelled to wear a head-dress composed of a thick circular pad, which was supposed to afford them some security against injury from falls; whilst strict limitations were placed upon their opportunities of exercise. On June 1st, 1839, the late Dr. Conolly took charge of the great asylum at Hanwell, and found the above described state of things existing there. From the 21st of the following September every form of mechanical restraint was absolutely discontinued in the institution. "The whole armoury," says one of his biographers, "of strait-waistcoats, straps, restraint chairs, &c., were laid aside." Together with these things, the bed-chains of the epileptics were relegated to a museum, and Conolly gave them firm pillows, of wedge shape, the thinner portion downward, so that if they turned over in bed there was no mechanical impediment to their respiration. Gradually their liberties were enlarged, they were encouraged to engage in various employments, and, within ten years, their benefactor was able to point to them as among the quietest and least troublesome of all the patients under his care. It was found that active bodily exertion at any suitable work, as in sawing wood or in digging, produced a notable diminution in the frequency and severity of their fits; as if the regular employment of nervous force in the accomplishment of orderly movement diminished the supply available for explosive and irregular discharges. The effect of these discharges upon the brain is always injurious; and the partial or complete cessation of the fits was found to be the sure precursor of general improvement.

While Conolly's great experiment was still in progress, the value of many of the compounds of bromine in the medical treatment of epilepsy was established by the late Dr. Brown-Sequard; and physicians, for the first time, found themselves in the possession of means by which the fits can, in many cases, be effectually controlled. From this time, at least in families sufficiently wealthy to afford every advantage to the subject of the disease, epilepsy has no longer been the terrible affliction which it was once rightly considered, although it would still be properly regarded as a disqualification for marriage, and as an insuperable bar to many forms of ordinary occupation.

Among the poor, however, the lot of epileptics has been far less effectually mitigated. An epileptic could not safely mount a ladder, or walk alone by the banks of a river. He could not be safely left in the vicinity of an open fire. For any kind of solitary work his usefulness is so conditioned and curtailed as to render it very difficult for him to find employment; and in collective work his position is scarcely better. The occurrence of a fit in a workshop would mean, almost certainly, the dismissal of the unfortunate epileptic. The practical outcome is that such a person cannot obtain work, or cannot keep it if obtained.

In the meanwhile, as Dr. Conolly had shown, the work which cannot be procured or cannot be safely undertaken, would be of all possible things the most salutary to the patient, would often suffice to control the fits without the aid of bromides, and would always be a potent adjuvant to these when they were required. A consideration of the facts led to the establishment of an Industrial Home for Epileptics at Bielefeld, in Westphalia; and the success of the experiment there made has induced benevolent persons in this country to establish a similar institution in England. The first steps were taken by a meeting at the Mansion House three or four years ago, a meeting at which the late Sir Andrew Clark advocated the project with more than his usual eloquence, and at which it was announced that the munificence of Mr. Passmore Edwards had supplied the promoters of the institution with the first essentials of its existence—land and buildings. It has been found at Chalfont St. Peter's, as at Bielefeld, that the epileptics derive immediate and striking benefit from the occupations which are open to them, and that physical work, carried to the point of fatigue, is of the highest efficacy in the prevention of paroxysms The whole of the inmates are engaged in productive labour, with a view to render the home as far as possible self-supporting, but a long time must of necessity elapse before very much can be accomplished in this direction. For the present, as a charitable institution, it is eminently deserving of support, for it seems to be incapable of "abuse" so long as care is taken that those admitted to share its benefits are not in a position to obtain similar advantages for themselves, or, if so, that they are charged remunerative terms for the benefit of the poorer inmates. Miss Twining, in a letter to the *Times*, has recently suggested that parochial authorities should be empowered to send epileptics to the home under proper conditions of payment; and it is manifestly only by some arrangement of this kind that its advantages can be extended to more than a fraction of the forty thousand epileptics who are to be found in England and Wales. In the meanwhile every contribution will be welcome; and the secretary, Mr. G. Penn Gaskell, will be glad to afford every information to inquirers. The London office is at 12, Buckingham Street, Strand.

